# The Importance of Lipidomic Approach for Mapping and Exploring the Molecular Networks Underlying Physical Exercise: A Systematic Review

**DOI:** 10.3390/ijms22168734

**Published:** 2021-08-13

**Authors:** Francesca Latino, Stefania Cataldi, Roberto Carvutto, Michele De Candia, Francesca D’Elia, Antonino Patti, Valerio Bonavolontà, Francesco Fischetti

**Affiliations:** 1 Department of Basic Medical Sciences, Neurosciences and Sense Organs, School of Medicine, University of Bari, 70123 Bari, Italy; francesca.latino@uniba.it (F.L.); stefania.cataldi@uniba.it (S.C.); roberto.carvutto@uniba.it (R.C.); michele.decandia@uniba.it (M.D.C.); francesco.fischetti@uniba.it (F.F.); 2 Department of Human Sciences, Philosophical and Education, University of Salerno, 84084 Fisciano, Italy; fdelia@unisa.it; 3 Department of Psychology, Educational Science and Human Movement, University of Palermo, 90128 Palermo, Italy; antonino.patti01@unipa.it

**Keywords:** biological systems, training, lipid profile, sports, metabolites

## Abstract

Maintaining appropriate levels of physical exercise is an optimal way for keeping a good state of health. At the same time, optimal exercise performance necessitates an integrated organ system response. In this respect, physical exercise has numerous repercussions on metabolism and function of different organs and tissues by enhancing whole-body metabolic homeostasis in response to different exercise-related adaptations. Specifically, both prolonged and intensive physical exercise produce vast changes in multiple and different lipid-related metabolites. Lipidomic technologies allow these changes and adaptations to be clarified, by using a biological system approach they provide scientific understanding of the effect of physical exercise on lipid trajectories. Therefore, this systematic review aims to indicate and clarify the identifying biology of the individual response to different exercise workloads, as well as provide direction for future studies focused on the body’s metabolome exercise-related adaptations. It was performed using five databases (Medline (PubMed), Google Scholar, Embase, Web of Science, and Cochrane Library). Two author teams reviewed 105 abstracts for inclusion and at the end of the screening process 50 full texts were analyzed. Lastly, 14 research articles specifically focusing on metabolic responses to exercise in healthy subjects were included. The Oxford quality scoring system scale was used as a quality measure of the reviews. Information was extracted using the participants, intervention, comparison, outcomes (PICOS) format. Despite that fact that it is well-known that lipids are involved in different sport-related changes, it is unclear what types of lipids are involved. Therefore, we analyzed the characteristic lipid species in blood and skeletal muscle, as well as their alterations in response to chronic and acute exercise. Lipidomics analyses of the studies examined revealed medium- and long-chain fatty acids, fatty acid oxidation products, and phospholipids qualitative changes. The main cumulative evidence indicates that both chronic and acute bouts of exercise determine significant changes in lipidomic profiles, but they manifested in very different ways depending on the type of tissue examined. Therefore, this systematic review may offer the possibility to fully understand the individual lipidomics exercise-related response and could be especially important to improve athletic performance and human health.

## 1. Introduction

It is now widely recognized that exercise is able to improve metabolic health of the whole body through a number of adjustments that occur at different levels [1]. These exercise-induced dynamic changes in tissue metabolites and lipids are the result of cellular and whole-body energy demands required in order to preserve metabolic homeostasis [2,3]. Living systems health is maintained by regulation of metabolic homeostasis due to several chemical reactions that emerge from different metabolic pathways and structures which constantly change the molecular environment of biological systems of the whole body. Physical exercise constitutes one of the toughest challenges to body energy homeostasis, because it requires continuous adaptive cellular stress responses to the ever-shifting internal and external environment [4,5,6,7,8]. However, the systemic regulation allowing homeostasis to be maintained in response to exercise is complex and remains poorly understood. In fact, although over the past decades huge progress had been made regarding the understanding of the cellular and molecular mechanisms involved in physical exercise through classical biochemical approaches, knowledge gaps still exist [9,10].

Fortunately, the continuous and rapidly increasing utilizations of “omics” technologies applied to physical exercise has offered new ways to analyze the complexity of biological networks at the basis of tissue-specific responses related to exercise [11]. Nowadays, they mark a major turning point in this research area. Mapping and exploring the molecular networks underlying physical exercise mainly involves omics approaches focused on lipids in a range of tissue samples and bodily fluids [12]. In response to modifications in body homeostasis induced by physical activity and exercise, the biological networks are stimulated in different cell types and organs in order to manage metabolic demands required to ensure that individuals adapt to new conditions imposed by exercise [13].

Lipidomics, which enables the study of complete lipid profile of an organism, plays a central role in the landscape of exercise-related metabolism. Studying the lipidomic profile allows the adjustments of the complex biological systems to be carried out more effectively in the context of exercise and their potential effect on promoting a broad variety of exercise-related health benefits [14]. The obtainable information through the lipidomic study depends on the observation compartment. Thus, the analysis of biological fluids (i.e., plasma) allows the evaluation of circulating lipids, such as triglyceride, phospholipids linked to proteins and fatty acids connected to cholesterol. On the other hand, the membrane lipidomic examines the composition of fatty acids of cell membrane [15].

As is well-known, the cell membrane is a cellular compartment that carries out many structural and functional tasks. The cell membrane is a bilayer phospholipids structure responsible for organizing of protein molecules. Its properties of fluidity and permeability depend largely on quality of fatty acids that form phospholipids [16]. Therefore, knowing the qualitative and quantitative balance of fats acids is crucially important in understanding organism responses to different stimuli offered by physical exercise. In this connection and in line with international research evidence, lipidomics is providing new and different possibilities to comprehend the role of lipids in biological regulations in response to physical exercise [17]. Lipidomics studies, in a dynamic way, the behavior of lipids from a structural and functional perspective in both physiological and pathological conditions [18]. It is strongly influenced by factors such as exercise, diet and diseases, thus providing a personal connotation thanks to a rationalization of the subjective metabolic situation [19]. Therefore, the lipidomic approach makes it possible to pinpoint and estimate lipid biomarkers underneath tissue-specific and systemic metabolic adaptations of human athletic performance [20].

Currently, lipidomic research tracks applied to human exercise responsiveness are significantly improved thanks to the use of targeted detection techniques, such as mass spectrometry (MS) and nuclear magnetic resonance (NMR) spectroscopy [21]. They allow exercise-induced adaptations to be assessed in the expression and activity of metabolic enzymes which have a bearing on an extensive range of biological functions. Several recent studies concerning this growing field [22,23] are mainly focused on lipid remodeling in skeletal muscle [24], since intramyocellular lipids also play a key part in athletic performance in relation with metabolic imbalances, in human blood [25], because through lipidomic analysis of the cell membranes of erythrocytes, it is possible to obtain an effective prevention on this molecular level, and in adipose tissue [26], due to the fact that exercise is able to increases glucose homeostasis, insulin sensitivity, and fatty acid oxidation.

Therefore, to elucidate the potential aspects of exercise biology that can benefit from the lipidomic approach, the purpose of this systematic review is to establish the biological action mechanisms of molecules eliciting the systemic effects in response to exercise. To this end, the most important lipidomic research paths over the last decade will be discussed, by providing a panel of the primary exercise-related lipid changes.

## 2. Results

### 2.1. Identification of Studies

**Figure 1:**
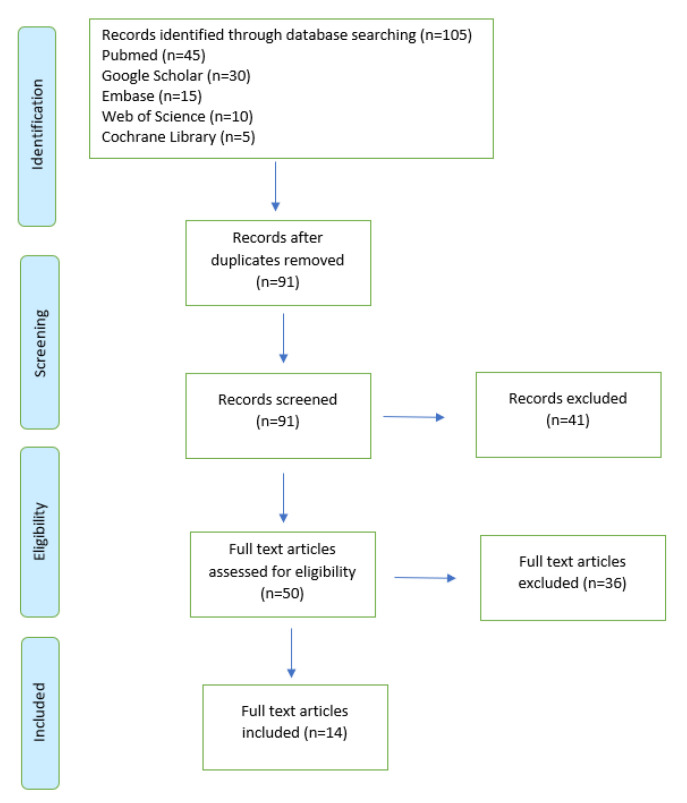
The study selection and eligibility screening flow according to PRISMA guidelines.

At the end of the selection process 105 articles were extracted, of which *n* = 45 from Pubmed, *n* = 30 from Google Scholar, *n* = 15 from Embase, *n* = 10 from Web of Science, and *n*= 5 from Cochrane Library. Each title and abstract were screened for relevance, removing reviews articles, unpublished studies, meta-analyses, case studies, practical guidelines and books (*n* = 41). Thereafter, the search strategy was based on the assessment of the full text of the remaining 50 articles in order to verify their eligibility. Lastly, 14 research articles specifically focusing on metabolic responses to exercise in healthy participants were included (Figure 1).

### 2.2. Study Characteristics

Thus, this systematic review provides an evaluation of 14 high-quality lipidomics-based studies [13,27,28,29,30,31,32,33,34,35,36,37,38,39] inquiring in depth about the exercise-induced changes of the human lipidome. Most of them (*n* = 10) [13,27,28,30,31,32,33,37,38,39] regarded acute lipidome alterations following intense and prolonged exercise, while the other works (*n* = 4) [29,34,35,36] focused on changes oncoming after a brief bout of acute exercise. Moreover, in 12 of these studies lipidomics was performed on biological fluids (blood and plasma); one was performed on skeletal muscle; and one collected both muscle biopsy and blood sample. Table 1 summarizes the characteristics of the studies included.

### 2.3. Outcome Measures of the Included Studies

Lipidomic alterations in response to exercise depend on the differed cell types, and intensity and duration of effort. The main outcome measures included in this analysis (85.7%; *n* = 12) measured lipidomic responses through the use of liquid or gas chromatography mass spectrometry (LC-MS; GC-MS) [13,27,28,29,30,31,32,33,34,35,36,38], ultra-performance liquid chromatography quadrupole time-of-flight mass spectrometry (UHPLC-Q-TOF-MS) in one study [37] and nuclear magnetic resonance (NMR) for one study [39].

## 3. Discussion

This systematic review aims to analyze and clarify the identifying biology of the individual response to different exercise workloads. Specifically, it provides an overview of the main lipidomes that are altered with both chronic and acute exercise training. To this end, the most important lipidomic research paths over the last decade were discussed, by providing a panel of the primary lipid changes exercise-related.

Lipidomics analyses of the studies examined revealed that medium- and long-chain fatty acids, fatty acid oxidation products, and phospholipids are subjected to different qualitative changes in response to exercise.

### 3.1. Lipidomic Analyses of Biological Fluids Following Exercise: The Blood Role

As previously discussed, exercise positively affects human metabolism in a number of biological fluids, which are constantly subject to adaptations in order to improve whole-body metabolic homeostasis in response to exercise itself [9]. Recently, lipidomics were used to examine these complex physiological responses to exercise [27]. This mutual relationship of beneficial influence between exercise and metabolism especially relates to blood cells [40]. Blood is considered a complete biofluid for a comprehensive lipidomic analyses thanks to the presence of metabolites exchanged with organs [41].

There are several studies that evaluated athletes’ lipidomic profiles in blood fluid, which proved that complex metabolic trajectories are highly adaptive and distinguishing differences between various groups of athletes were available, for instance with respect to gender, age-specific data and intensity of exercise [42]. Exercise lipidomics performed on the blood can give precious information concerning the end biological impact and biological processes associated with acute exercise as well as endurance performance [43].

These approaches are employed to estimate adjustments in metabolites connected to lipid metabolism at variable exercise intensities and durations in body fluids such as blood and plasma [44]. A number of studies have proved that metabolomics and lipidomics may bring to light disorders in oxidative stress [45], abnormalities in energetic substrates used during physical exercise [46], and various metabolic phenotypes related to physiological parameters (i.e., VO_2max_ and lactate clearance capacity) [47].

Exercise lipidomics can map out an abundance of lipid species and metabolites that change in a dynamic way in response to metabolic stresses exercise-induced [48]. Specifically, plasma metabolites are capable of addressing both the metabolism of a certain organ and modifying whole-body metabolism [49]. Thus, investigating lipidomic profiles associated with training status may have a prognostic value, as well as it may help to develop exercise interventions based on athletes’ lipidomic profiling.

Nieman, Sha, and Pappan [31] measured metabolite responses in both muscle biopsy and plasma samples collected pre- and post-long-duration, high-intensity running. They showed a 33.7 ± 4.2% decrease in muscle glycogen, 39.0 ± 8.8-, 2.4 ± 0.3-, and 1.4 ± 0.1-fold increases in plasma IL-6, IL-8, and monocyte chemoattractant protein-1, respectively, and 95.0 ± 18.9 and 158 ± 20.6% increases in cortisol and epinephrine, respectively. Lipidomics analysis detected 209 metabolites modified as a consequence of exercise. These metabolites were especially represented by high levels of long and medium-chain fatty acids, fatty acids oxidation products, and ketone bodies. Moreover, in contrast with previous studies, the authors found that the increase in plasma IL-6 following long endurance running has a minor relationship compared to increases in lipid related metabolites.

Manaf et al. [32], rated 18 healthy males engaged in long-duration, high-intensity cycling activity in order to identify the changes in the metabolic profile of plasma after fatigue of the exercise. They identified 68 metabolites changed at the end of exercise and found that the metabolites that played a central role in contributing to induce fatigue post-exercise were free-fatty acids (FFA) and tryptophan (TRP). Their findings showed a highest increase in oleic acid, linolenic, and palmitic acids, as well as in the levels of oleoylcarnitine, palmitoylcarnitine, and acetylcarnitine. These metabolites seem to play a key role during exercise-induced fatigue onset.

In a 2020 study, Varga et al. [37] investigated blood lipidomic profiles in female elite endurance athletes. They showed how blood lipid trajectories changed in response to intensive exercise and that these adaptations were related with different cardiometabolic, endocrine, bone and energy comorbidities associated with Relative Energy Deficiency in Sport (RED-S). In their study, findings revealed that long-chain fatty acids, such as TG(47:1), TG(51:1), and TG(55:3), triacylglycerols that increase in response to the exercise interventions in participants with eumenorrhea. The same response was found in the level of SM(d41:1), a sphingomyelin that demonstrates a large reactivity to the exercise interventions.

While these studies faced mainly the metabolic changes occurring in plasma, serum, or whole blood, only a few research studies have concentrated on metabolic modifications in red blood cells (RBCs) especially with regard to the type and level of physical exercise. Based on the assumption that erythrocytes represent the greatest part of circulating blood cells, Nemkov et al. [38] hypothesized that the metabolic state of red blood cells would result in being correlated with endurance capacity. With a view to develop a greater comprehension of the metabolic environment related with changes in red blood cells in response to exercise, their research meant to evaluate the effects of high-intensity, prolonged physical exercise on concentrations of the lipid markers in various blood components. They found that acute high-intensity exercise determines oxidative stress and changes in free fatty acids that causes lipid damage in red blood cells with consequent membrane lipid remodeling. Thus, their results revealed that the levels of alanine were higher at baseline and decreases after cycling in conjunction with increased pyruvate and tricar-boxylic acid (TCA) cycle metabolites, as result of an increased energetic demands. At the same time, increased levels of dodecanoic and tetradecanoic acid allowed the release of free fatty acids, which are conjugated to carnitine for mitochondrial import and oxidation. Moreover, they observed high levels of acetylcarnitine and propionylcarnitine during cycling, as well as the tryptophan catabolites in-dolequinone-carboxylate and È-oxalocrotonate also increased ɣ-oxalocrotonate.

In the same way, Gollasch, Dogan, Rothe, Gollash, and Luft [35] assessed the impact of acute exercise on individual red blood cell fatty acids in healthy subjects undergoing maximal treadmill exercise. The authors measured the plasma levels of epoxides derived from cytochromes P450 (CYP) monooxygenase and lipoxygenase (LOX), mediators derived from both the n-3 and n-6 fatty acid (FA) in healthy volunteers before, during and after short-term exhaustive exercise. Their findings demonstrated that exhaustive exercise increased the circulating levels of epoxyoctadecenoic (12,13-EpOME), dihydroxyeicosatrienoic (5,6-DHET), dihydroxyeicosatetraenoic acids (5,6-DiHETE, 17,18-DiHETE). On the other hand, no changes were found in the levels of omega-3 fatty acids in the systemic circulation. These findings were consistent with the idea that the essential fatty acids (n-3 and n-6) are deeply affected by food intake [50], but not short-term maximal exercise, whereas changes in red blood cell lauric acid reflects metabolic processes. Concerning that, more research is needed to determine the contribution of erythrocyte fatty acids to physical performance in health and cardiovascular disease. Concerning the prognostic value of the lipidomic, an increasing number of studies analyzed the impact of exercise-induced changes on human metabolites under both physiological and pathological status [14,51]. This research used various training interventions and study designs and conducted metabolomics and lipidomics in individuals with a variety of health conditions. The search has reported different alterations in metabolites linked to oxidative stress, and fatty acid metabolism. These findings have resulted in a deeper knowledge of the effects of physical activity in biological systems. Shining examples are modifications that occur at the end of exercise in plasma fatty acids, fatty acid oxidation products, and minor phospholipids. Specifically, metabolomics and lipidomics are able to increase scientific background of the significant, although complex, impact that physical activity has on the related molecular mechanisms in patients with pathological conditions [52,53].

Jastrzebski et al. [39], in their recent work, analyzed lipid profiles of 14 patients with sarcoidosis. Their results showed that lipid profiles were substantially altered after a 3-week exercise training program. They included decreases in fatty acids, triglycerides, and total cholesterol. Other alterations involved shifts in fatty acids oxidation products and triacylglycerol esters.

These findings were supported from the significant modifications in lipid profiles confirmed by lipidomics. Given that, it is possible to argue that resistance exercise program may have beneficial effects on exercise tolerance, serum proinflammatory cytokine levels, and lipid profiles, which represent important risk factors for the occurrence of sarcoidosis complications. The effectiveness of these exercise-induced alterations in lipid profiles represents a valuable prognostic method. Wang, Shen, and Xu [36], stated that aerobic exercise for 8 weeks shown to have beneficial effects on plasma lipids by leading a significant decrease in serum concentrations in patients with coronary heart disease. Nevertheless, Fikenzer et al. [54] argued that only endurance training performed 3–4 days a week for 40–50 min per session over a period of 26 weeks is able to lead improvements in serum lipid levels.

Altogether, these studies allow to illustrate the power of a deep analysis of lipidomic profile to be illustrated in order to ensure understanding of the complex physiological response of humans to exercise, pinpoint the main biological processes implicated in maintenance of the healthy status and establish about impaired mechanisms associated with some pathological conditions.

### 3.2. Exercise-Related Lipidomics Analysis in Muscle Tissue

A superb observing point from which to analyze lipidomics in response to physical exercise is skeletal muscle cell membrane. It represents a cellular compartment with numerous and well-known structural and functional tasks, which has increasingly come to the fore because of its role as a metabolic pacemaker [55]. In fact, the arrangement of the cell membrane and fatty acids stored in it clearly defines the regulation of whole cellular activity, signals picked up for the initiation of metabolic pathways, until their impact on pathological status. In recent years, it was clarified how these mechanisms may be affected in a decisive way by environment and lifestyle of individuals and how just the cell membrane plays the central role in collecting and transferring process of regulatory information [56].

Lipids in skeletal muscle are of paramount importance for the maintenance of energy resources involved in muscle metabolism. The effectiveness of exercise on improving skeletal muscle metabolism has been well-confirmed [57]. Physical exercise activates complex molecular responses that involve significant changes in metabolic pathways (such as glycolysis and fatty acid oxidation), as well as in acute inflammatory markers (such as interleukin-6) [58].

Physical exercise determines disruption in whole-body homeostasis, thus each cell and organ have to face and adapt to greater metabolic, mechanical, and thermoregulatory demands combined with a higher amount of work [59]. Indeed, as a result of the augmented requirement in substrates in the course of the acute or chronic physical exercise, lipidomic profiles will be helpful to establish the lipid metabolite features of the muscles exposed to exercise.

Thus, lipidomics related to exercise-responsive factors provide significant information about the status of skeletal muscle to modify whole-body glucose and lipid homeostasis [60]. In this regard, a deeper lipidomic analysis provides a better understanding of these mechanisms and may enhance knowledge of exercise physiology and address the practice of exercise towards the treatment of certain pathologies. After all, skeletal muscle is the crucial tissue that contributes to bodily energy metabolism, including insulin-dependent glucose uptake and lipid oxidation [61]. The fatty acids composition of the skeletal muscle membrane is correlated to peripheral response related to insulin and obesity, which are both susceptible to physical exercise. Overall, it was detected that an increase in fatty acids unsaturation of the skeletal muscle membrane in response to exercise is linked to an improvement of the insulin sensibility [62]. In fact, the composition of membrane phospholipids is a dynamic system, whose mechanisms are not completely understood. The concomitance of both genetic and lifestyle factors, including diet and physical activity, would seem to play a key role in the determination of the composition of muscle membrane phospholipids [63]. The value of performing lipidomic profiling was recently highlighted in a study that identified divergent lipidomic adaptations in white and brown adipose tissue from mice subjected to exercise training [64].

In this context, lipidomic analyses allow the status of the cell membrane of the skeletal muscle to be highlighted, which is connected to the status of other membranes and with individual lipid metabolism, which is in turn linked to healthy status, lifestyle, nutrition, and general metabolic condition [65]. The purpose of this examination is to provide an overview of the situation of the athlete, namely a personalized analysis of the individual lipidomic condition. Indeed, the fatty acids composition is particular for each tissue so that the skeletal muscle membrane will have its own distinctive percentage distribution of fatty acids. It is somewhat highly significant since it presents both lipids synthesized at an endogenous and exogenous level. Therefore, a shift from normal value may indicate, also in a preventive way, a situation of cellular suffering, resulting in an unbalance of proportional ratios among the various fatty acids families [51]. In a study conducted by Andersson, Nälsén, Tengblad, and Vessby [66], it emerged that the composition of the fatty acids muscle membrane phospholipids in both healthy men and women was influenced by diet, as much as physical exercise. These findings confirmed the impact that regular physical exercise may have on the maintenance of the right balance among different phospholipid families. Several studies in this area showed that exercise is a potential mediator of this stability. In this respect, in 1998, Anderson, Sjödin, Olsson, and Vessby [67] demonstrated for the first time that 6 weeks of moderate-intensity physical exercise determined significant changes in fatty acids composition which constitute muscle phospholipids membrane, resulting in a meaningful increase in oleic acid, and a decrease in aminoacids. In an important study, Goto-Inoue and co-workers [27] verified the characteristic lipid species in skeletal muscle of a chronic exercise training compared it to high-fat-induced obesity. They reported a significant fatty acid remodeling of phospholipids in response to chronic training that can alter lipid metabolism in skeletal muscle, by resulting from insulin sensitivity and resistance [68]. Specifically, linoleic acid-containing phosphatidylcholine and sphingomyelin and docosahexanoic acid-containing phosphatidylcholine were identified as chronic exercise training-induced lipids, while arachidonic acid-containing phosphatidylcholines, phosphatidylethanolamines, and phosphatidylin-ositol were associated with high-fat diet-induced lipids. Lastly, minor sphingomyelin, which has long-chain fatty acids, was a high-fat diet-specific lipid. In recent years, the related and emerging literature that has seen a growing interest in investigating the relationship between the application of lipidomic and exercise-related inflammation. Several reports on this relationship found that exercise increases inflammation in the early recovery phase [69] and decreases polyunsaturated fatty acids with anti-inflammatory and protective activity [70,71]. Approximately 2 h after the end of exercise, considerable increases in various lipid mediators were observed in skeletal muscle. They included cyclooxygenase (COX), prostaglandins (PGs), thromboxanes (TXs), and derived from arachidonic acid (AA). Moreover, efforts in this field have observed that significant differences in lipidome of the skeletal muscle are presented in relation to individual’s age [72]. Specifically, these changes are represented by an increase in older men of ceramides, a subclass of sphingolipids which covers the role of activators of proinflammatory signaling [73,74,75]. They showed markedly uppermost palmitic (C16:0) and arachidic (C20:0) ceramides levels in the skeletal muscle. These results indicated that the existence of higher intramuscular levels of certain ceramides could have negative effects on anabolic signaling on completion of a session of resistance exercise by fostering inflammation [76,77,78]. Thus, in view of the above, lipidomics allows us to capture the state of muscle skeletal cell membrane and learn about its needs in order to better verify its equilibrium and early identify possible deficiencies on which to intervene.

### 3.3. Lipidomic Profile in Acute and Chronic Physical Exercise

In the last past decade, thanks to the progress that has been achieved as far as mass spectrometry is concerned, an increasing number of lipidomic-based studies investigated the lipid trajectories correlated to different types of exercise [5,7,79,80,81,82]. In addition, bioinformatics provided strong support, as it is able to improve the overall understanding of the body’s lipidome response to exercise [83]. These techniques of lipidomic profiling enable the exact nature of exercise-responsive lipids that can influence whole-body homeostasis to be identified. This area of the literature focused on basic concepts behind the diverse metabolic responses to varying acute and chronic physical exercise. Throughout this perspective, lipidomic analysis allow the detection of the distinct impact on the metabolic and molecular responses of any given tissue based on different frequency, intensity, and duration of each type of exercise [84]. An increasing number of scientific publications in this field indicated that a bout of prolonged and intensive exercise is able to determine huge changes in multiple lipid-related metabolites [85,86,87,88,89,90]. Long-term participation in vigorous exercise programs determines in athletes relevant increases in more than 300 metabolites [91]. In this connection, the most solid alterations following endurance exercise regard lipid metabolism. Specifically, it was possible to observe a substantial increase in plasma medium- and long-chain fatty acids, fatty acid oxidation products, and acylcarnitines, accompanied by a decrease in phospholipids [92]. These responses occur in a window of time ranging from a few hours after prolonged and intensive exercise to a day of recovery after training. The exercise-related changes in response to heavy exercise workloads are the result of the physiological stress induced by training [93].

Karl and colleagues [30] evaluated metabolic responses to extreme physiologic stress induced by a military endurance exercise. At the end of their study, they found changes in multiple metabolites (n = 478 metabolites changed from pre- to post-exercise) related to lipid metabolism. They involved an increase in acylcarnitines, energy metabolism, lipolysis, fatty acid oxidation, ketogenesis, and branched-chain amino acid catabolism. On the contrary, they observed a decrease in mono- and diacylglycerols.

Similarly, Nieman, Shanely, Gillitt, Pappan, and Lila [28] assessed serum metabolic signatures induced by a three-day intensified exercise period. They documented that their athletes experienced a deep systemic alteration in blood metabolites related to energy production especially from the lipid pathway following 3 days of heavy exertion. Indeed, at the end of exercise, significant increases in 75 metabolites were identified. The most of these metabolites related to lipid/carnitine metabolism, 13 to amino acid/peptide metabolism, 4 to hemoglobin/porphyrin metabolism, and 3 to Krebs cycle intermediates. After a 14 h recovery period, 50 metabolites remained elevated, while 8 decreased (primarily amino acid-related metabolites). Significant decreases (40–70%) in 22 metabolites related to lysolipid and bile acid metabolism were found following exercise.

Howe and co-workers [33], in a study where they simulated a treadmill ultramarathon, investigated nine runners’ metabolomic profile. Using high-resolution mass spectrometry, they showed identified 446 metabolites changed following exercise. Specifically, their study revealed that fatty acid metabolism was affected after training, with an increase in the formation of medium-chain unsaturated and partially oxidized fatty acids (i.e., linoleic acid 9-hydroxylinoleic acid and 13-hydroxylinoleic acid) and conjugates of fatty acids with carnitines. At the same time, they observed several amino acids decreased post-80.5 km.

Concerning acute bouts of exercise, there are comparatively few studies which analyzed exercise-induced lipidome changes [94,95,96]. In comparison to prolonged exercise and high-volume workloads, these studies documenting those alterations in lipidome levels were present in both exercises, but they manifested in much different ways. In a 2018 study, Al-Khelaifi et al. [34] compared the metabolic profiles of elite-level athletes from different sporting disciplines. Their findings showed that high-power and high-endurance athletes presented different metabolic profiles with regard to fatty acid metabolism, oxidative stress, and energy-related metabolites. In particular, gamma-glutamyl amino acids were significantly reduced in both high-power and high-endurance athletes. High-endurance athletes showed decrease in diacylglycerols and ecosanoids. On the contrary, high-power athletes exhibited increased levels of phospholipids and xanthine metabolites. This study is representative because it reflects the potential that metabolomics and lipidomics offer when applied to exercise. Indeed, they enable a quantitative analysis of the metabolic profiles with the objective of identifying several biological factors associated with physical performance, such as response to fatigue, sports-related disorders, and alterations in response to pre- vs. post-exercise [97,98,99]. Likewise, Peake et al. [29] emphasized the importance of lipidomic analysis to provide extensive information about the metabolic demands of exercise. In their study, they compared lipidomic profile of high-intensity interval training with moderate-intensity continuous exercise. After intervention they found a significant increase in monounsaturated fatty acids in response to high-intensity interval training. They identified 49 metabolites, among which 11 were altered following both high-intensity interval training (HIIT) and moderate-intensity continuous exercise (MOD), 13 changed after HIIT, and five changed after MOD. Significant alterations involved increases in tricarboxylic acid intermediates and monounsaturated fatty acids after HIIT. Moreover, they found important decreases in amino acids during recovery from both training.

Contrepois et al., in a relevant 2020 work [13], proved a significant increase in circulating complex lipids which included 23 phosphatidylcholines, 20 cholesteryl esters, 15 triacylglycerols, 10 diacylglycerols, nine ceramides and eight sphingomyelins. They occurred 2 min after cardiopulmonary exercise test (CPX) test and then returned to pre-exercise value following 15–30 min. They showed that plasma concentrations of triacylglycerols (TAGs), specifically those with shorter saturated fatty acids, decreased after approximately 30 and 60 min of recovery. On the contrary, long-chain polyunsaturated fatty acids (PUFA) including arachidonic acid (AA), eicosapentaenoic acid (EPA) and decosahexaenoic acid (DHA) rapidly increased within 2 min in response to exercise. These alterations in long-chain PUFA were consistent with a trigger of pro- and anti-inflammatory pathways [100,101].

Evaluating metabolomic and lipidomic signatures associated with athlete’s performance allows potential pathways to be identified that differentiate endurance exercise from acute exercise [102,103,104]. Thus, learning about these changes can help to provide a prominent and complete overview of the physical condition of athletes, as well as their adaptation to training, in order to enhance their athletic performance.

## 4. Methods

### 4.1. Search Strategy

This systematic review was carried out following the PRISMA statement [105]. Two author teams performed initial research question, which were then transformed according to the participants, intervention, comparators, outcomes, and study design (PICOS) system. The databases used for the identification of scientific articles were Medline (PubMed), Google Scholar, Embase, Web of Science, and Cochrane Library. Candidate studies were identified by using the following Boolean search syntax: “((lipidomes or lipidomic) and (exercise or “physical exercise”)”/“(metabolites or metabolomic) and (training or “physical training”)”/“(“omics approach” or “biological path”) and (exercise or training))”. Afterwards, the following filters were activated—text availability: full text; species: humans or animals; languages: English. The search strategy used for the PubMed database was a combination of the MeSH database and Boolean search syntax. Conversely, the search syntax was adapted appropriately for searching the Web of Science. After candidate articles were collected, further identification was conducted based on inclusion and exclusion criteria.

### 4.2. Selection Criteria

In this systematic review, studies were considered eligible for inclusion if they provided relevant information on PICOS system and met the following inclusion criteria:

- English-language publications;
- Time interval of studies between 2010 and 2021;
- Inclusion of clear measures of a lipidomic profile related to exercise;
- Study design: randomized controlled trials with pre-and post-measures;
- Intervention: all types of physical activity.

**Figure 2:**
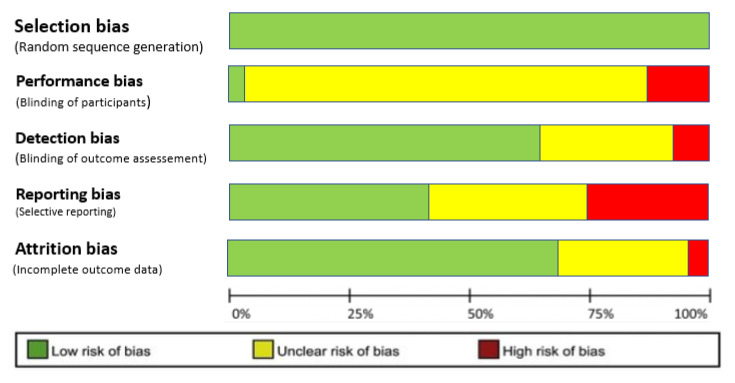
Risk of bias graph of included studies. The colors represent the quality of the studies: red = high risk, yellow = uncertain, green = low risk.

Studies were excluded if they did not meet the above criteria, or due to the lack of focus on exercise-lipidome changes. Review articles, meta-analysis, and unpublished studies were excluded from this work, although they were used as a reference to identify the original search to examine for inclusion. The modified version of the Jadad Scale was used to select the studies in a qualitative way (Table 2) and assess risk of bias (Figure 2).

## 5. Concluding Remarks

The ever-increasing application of lipidomic-based technologies constitutes an exceptional way to enhancing the knowledge of the complex relationship between exercise and related metabolic adaptations. In fact, exercise induces changes in a high number of metabolites, especially after prolonged and intensive exercise.

Despite the fact that it is well-known that lipids are involved in different sport-related changes, it is unclear what types of lipids are involved. Therefore, in this systematic review, we analyzed the characteristic lipid species in blood and skeletal muscle, as well as their alterations in response to chronic and acute exercise. Lipidomics analyses of the studies examined revealed lipid qualitative changes. Specifically, the major changes concern medium- and long-chain fatty acids, fatty acid oxidation products, with a large elevation in acylcarnitine levels, phospholipids, linoleic acid-containing phosphatidylcholine and sphingomyelin and docosahexanoic acid-containing phosphatidylcholine as result of chronic exercise training-induced lipids. Conversely, long-chain polyunsaturated fatty acids (PUFA) including arachidonic acid (AA), eicosapentaenoic acid (EPA) and decosahexaenoic acid (DHA) were characterized as acute exercise training-induced lipids. Fatty acid oxidation was stimulated by exercise as result of accumulation of many acylcarnitines and free fatty acids. The impact of exercise on carnitines has been observed in most of these studies. A possible explanation is that the carnitines reflect mitochondrial fatty acid oxidation as an energy source under the impact of exercise. Among the studies analyzed, exercise was also characterized by an accumulation of different complex lipids including cholesteryl esters, phosphatidylcholines, diacylglycerols, ceramides and sphingomyelins. Sphingolipids, and in particular ceramides, were mainly involved in inflammation in response to exercise. In exactly parallel fashion, associated drop occurs in minor phospholipids such as lysophosphatidylcholines and lysophosphatidylethanolamines. These metabolites were especially located on the molecular layer of both blood and muscle cell membrane and their distribution differed among cell types, leading to different types of signal transduction within cells. Changes in lipid profiles from red blood cells included complex lipid, epoxy FA, AA, EPA, DHA, and n-3 and n-6 PUFA. While changes in lipid profiles within muscle skeletal mainly regarded significantly higher acylcarnitines, palmitic (C16:0) and arachidic (C20:0) ceramides levels, and release of medium-chain FA.

The findings from this review may offer the possibility to fully understand the individual response to exercise in order to extend exercise’s molecular landscape and promote benefits on an individual level. Therefore, this systematic review offers an overview of the lipidomes that are altered with exercise training and could be especially important to improve athletic performance and human health. Indeed, the translation potential of the study resides in the valuable analysis of the athletes’ current physical status and their adaptation to training. Nevertheless, this frontier in exercise biology is still emerging, and much remains to be discovered, specifically how certain biological pathways are recruited and regulated in response to the variations in exercise timing, intensities, training programs, and different sporting disciplines.

Future lipidomic-based exercise training studies should continue controlling the biological response to acute and prolonged exercise. It would be helpful that future research will route its efforts towards the understanding of the exercise-related response in both men and women at a variety of ages and levels, and type of exercise. Moreover, future research will better define lipidomic responses to compare the effects in response to HIIT compared to MOD.

## Figures and Tables

**Table 1:** Summary characteristics of reviewed studies.

Authors	Sample	Research Design	Variables of the Study	Results
Goto-Inoue et al. (2013)	Trained and untrained rats	6 weeks of Chronic exercise associate with 12 weeks of high fat diet	muscle tissues samples	The findings reveal compositional changes in phospholipid molecular species: ↑ linoleic acid-containing phosphatidylcholine and sphingo-myelin, ↑ docosahexanoic acid-containing phosphatidylcholine
Nieman et al. (2013)	35 long-distance male runners (supplemented group: aged 33.7 ± 6.8 years; placebo: aged 35.2 ± 8.7 years)	3-day intensified exercise (2.5 h at 70%VO_2max_ bouts)	blood samples: pre- and post-14-day supplementation of polyphenol-enriched protein, and immediately and 14 h after the 3rd day of training	324 metabolites changed: ↑ metabolites related to fatty acid oxidation and ketogenesis including free fatty acids, acylcarnitines, 3-hydroxy-fatty acids, and dicarboxylic acids, amino acid and carbohydrate metabolism.
Peake et al. (2014)	10 well-trained male cyclists and triathletes (aged 33.2 ± 6.7 years)	Moderate-intensity vs. continuous exercise	blood samples: pre- and post-exercise	49 metabolites identified: Significant increase in monounsaturated fatty acids in response to high-intensity interval training, ↑ in carbohydrate oxidation and ↓ in fat oxidation
Karl et al. (2017)	25 male highly trained soldiers (aged 19.0 ± 1.0 years)	Military endurance exercise	blood sample: pre- and post-exercise	478 Metabolites changed pre- and post-exercise: ↑ free fatty acids; ↑ acylcarnitines; ↓ mono- and diacylglycerols; ↑ branched chain amino acid metabolites
Nieman et al. (2017)	24 trained male runners (aged 36.5 ± 1.8 years)	One bout run to exhaustion at 70%VO_2max_	Muscle biopsy and blood samples collected before and immediately after running	209 metabolites altered: ↑ long and medium-chain fatty acids, ↑ fatty acids oxidation products (dicarboxylate; monohydroxy fatty acids; acylcarnitines), ↑ ketone bodies. Minor relationship with ↑ IL-6.
Manaf et al. (2018)	18 healthy and recreationally active males (aged 24.7 ± 4.8 years)	Cycling test at a workload 3 mM/L lactate	blood samples: pre-exercise, during exercise (10-min, before fatigue), exhaustion point, post-exercise (20 min after fatigue)	68 metabolites changed: ↑ Free-fatty acids and ↓ tryptophan (fatigue).
Howe et al. (2018)	9 male ultramarathon runners (aged 34 ± 7 years)	80.5 km treadmill simulated ultramarathon run	blood samples: pre- and post-exercise	446 metabolites identified:↓ amino acids metabolism post-80.5 km; ↑ in the formation of medium-chain unsaturated, partially oxidized fatty acids
Al-khelaifi et al. (2018)	191 elite athletes (171 males, 20 females)	Elite athletes from various sport disciplines	blood samples collected IN or OUT competition	High-power athletes exhibited increased levels of phospholipids compared to high-endurance athletes
Gollasch et al. (2019)	6 healthy volunteers (five male and one female); (aged 38 ± 15 years)	Maximal treadmill exercise	blood samples collected pre-exercise, during exercise and post-exercise	Exhaustive exercise increased the circulating levels of epoxyoctadecenoic, dihy-droxyeicosatrienoic, dihydroxy-eicosatetraenoic acids. Exercise does not change the levels of omega-3 fatty acids in the systemic circulation
Wang et al. (2019)	38 patients with coronary heart disease (19 untrained + 19 trained)	Aerobic exercise for 8 weeks	blood samples collected pre- and post-exercise	Findings shown beneficial effects on plasma lipids by leding a significant decrease in serum concentrations in patients with coronary heart disease. Significant ↓ in triglyceride and apoC3 concentration; significant ↑ in HDL-C
Contrepois et al. (2020)	36 men (aged 40–75 years)	CPX testing and serial blood collection	blood samples collected before exercise (baseline) as well as 2 min, 15 min, 30 min, and 1 h in recovery.	Lidip analysis indicates: ↑ acylcarnitines, free fatty acids, complex lipids, and amino acids
Varga et al. (2020)	38 female elite endurance athletes (aged 18–38)	Athletes underwent a day-long exercise test	blood samples drawn five times: at fasting state, andbefore and after two separate exercise tests in the morning and in the afternoon	Lidip analysis indicates that participants with menstrual dysfunction might have decreased adaptive response to exercise intervention. Lipid trajectorie altered: Glycerolipids, Glyceropho-spholipids, Sphingo-lipids
Nemkov et al. (2021)	8 well-trained male athletes (aged 35 ± 8)	30 min high-intensity cycling test	blood samples collected before and 3 min after a 30 min submaximal cycling test	Acute high-intensity exercise determines oxidative stress and changes in free fatty acids that causes lipid damage in red blood cells. Results revealed an increased levels of dodecanoic and tetradecanoic acid that indicate ongoing lipolysis to release free fatty acids, conjugated to carnitine
Jastrzebski et al. (2021)	14 patients with sarcoidosis (aged 46.0 ± 9.6)	Moderate intensity exercise training	blood samples collected after overnight fasting at baseline and after the 3-week exercise training	Lipid profiles were altered after 3 week exercise training program decreases in fatty acids, triglycerides and total cholesterol. Other changes included shifts in fatty acids oxidation products and triacylglycerol esters

**Table 2:** The modified Oxford quality scale.

Authors	Was the Treatment Randomly Allocated?	Was the Randomization Procedure Described and Was Appropriate?	Was There a Description of Withdrawals and Dropout?	Was There a Clear Description of the Inclusion/Exclusion Criteria?	Were the Methods of Statistical Analysis Described?	Jadad Score (0–5)
Goto-Inoue et al. (2013)	Yes	Yes	No	No	Yes	3
Nieman et al. (2013)	Yes	Yes	Yes	Yes	Yes	5
Peake et al. (2014)	Yes	Yes	No	Yes	Yes	4
Karl et al. (2017)	Yes	Yes	No	No	Yes	3
Nieman et al. (2017)	Yes	Yes	Yes	Yes	Yes	5
Manaf et al. (2018)	Yes	Yes	No	Yes	Yes	4
Howe et al. (2018)	Yes	Yes	No	No	Yes	3
Al-khelaifi et al. (2018)	Yes	Yes	No	Yes	Yes	4
Gollasch et al. (2019)	Yes	Yes	No	Yes	Yes	4
Wang et al. (2019)	Yes	Yes	No	Yes	Yes	4
Contrepois et al. (2020)	Yes	Yes	No	No	Yes	3
Varga et al. (2020)	Yes	Yes	No	No	Yes	3
Nemkov et al. (2021)	Yes	Yes	No	No	Yes	3
Jastrzebski et al. (2021)	Yes	Yes	Yes	yes	Yes	5

## Data Availability

The data presented in this study was obtained from the included studies and was openly available.
